# Biosynthesis of Polyunsaturated Fatty Acids in the Oleaginous Marine Diatom *Fistulifera* sp. Strain JPCC DA0580

**DOI:** 10.3390/md11125008

**Published:** 2013-12-11

**Authors:** Yue Liang, Yoshiaki Maeda, Yoshihiko Sunaga, Masaki Muto, Mitsufumi Matsumoto, Tomoko Yoshino, Tsuyoshi Tanaka

**Affiliations:** 1 Division of Biotechnology and Life Science, Institute of Engineering, Tokyo University of Agriculture and Technology, 2-24-16, Naka-cho, Koganei, Tokyo 184-8588, Japan; E-Mails: 50011702110@st.tuat.ac.jp (Y.L.); y_maeda@cc.tuat.ac.jp (Y.M.); 50010702105@st.tuat.ac.jp (Y.S.); m_muto@cc.tuat.ac.jp (M.M.); y-tomoko@cc.tuat.ac.jp (T.Y.); 2 Japan Science and Technology Agency (JST), Core Research for Evolutionary Science and Technology (CREST), 5, Sanbancho, Chiyoda-ku, Tokyo 102-0075, Japan; E-Mail: Mitsufumi_Matsumoto@jpower.co.jp; 3 Biotechnology Laboratory, Electric Power Development Co., Ltd, 1, Yanagisaki-machi, Wakamatsu-ku, Kitakyusyu 808-0111, Japan

**Keywords:** marine oleaginous diatom, *Fistulifera* sp. JPCC DA0580, eicosapentaenoic acid, polyunsatulated fatty acid, desaturase

## Abstract

Studies of polyunsaturated fatty acid (PUFA) biosynthesis in microalgae are of great importance for many reasons, including the production of biofuel and variable omega 3-long chain PUFAs. The elucidation of the PUFA biosynthesis pathway is necessary for bioengineering to increase or decrease PUFA content in certain microalgae. In this study, we identified the PUFA synthesis pathway in the oleaginous marine diatom, *Fistulifera* sp. strain JPCC DA0580, a promising candidate for biodiesel production. The data revealed not only the presence of the desaturases and elongases involved in eicosapentaenoic acid (EPA) synthesis, but also the unexpected localization of ω3-desaturase expression in the chloroplast. This suggests that this microalga might perform the final step of EPA synthesis in the chloroplast and not in the endoplasmic reticulum (ER) like other diatoms. The detailed fatty acid profile suggests that the EPA was synthesized only through the ω6-pathway in this strain, which was also different from other diatoms. Finally, the transcriptome analysis demonstrated an overall down-regulation of desaturases and elongases over incubation time. These genetic features might explain the decrease of PUFA percentage over incubation time in this strain. The important insights into metabolite synthesis acquired here will be useful for future metabolic engineering to control PUFA content in this diatom.

## 1. Introduction

Bioenergy production (e.g., bioethanol, biobuthanol, and biodiesel) using photosynthetic systems is widely accepted as a promising solutions to the increasing energy crisis. Among biofuels, biodiesel fuel (*i.e*., fatty acid methyl ester: FAME) production using microalgal biomass is an emerging technology, and has attracted increasing attention due to advantageous features for a sustainable fuel supply (e.g., capacity for CO_2_ fixation, independence from agriculture, and higher yield of biofuel raw materials as compared to land plants) [1].

In biodiesel production from microalgal metabolites, FAMEs are generated by methanolysis of microalgal lipids (mainly triacylglycerols and perhaps a few free fatty acids), and the FAME composition directly determines the biodiesel fuel quality. The physical and chemical properties of the FAMEs depend on the structural features of the acyl chain, e.g., carbon chain length, chain branching, and unsaturation degree. For example, the high proportion of polyunsaturated fatty acid methyl esters (PUFAMEs) will lead to less oxidative stability of the final biodiesel [2], and, thus, have been limited to less than 1% by the EN 14214 standards in Europe. Specific enzymes, *i.e.*, desaturases and elongases are responsible for fatty acid desaturation and elongation reactions. Molecular engineering strategies have been proposed to suppress the expression of desaturase genes, to reduce the proportion of polyunsaturated fatty acids (PUFA), with some successful attempts in decreasing the unsaturation degree of lipids in terrestrial plants, such as soy beans [3,4,5]. However, little progress has been reported in microalgae despite tremendous efforts. This may be attributed to insufficient knowledge regarding PUFA synthesis in microalgae, except for that in some model organisms [6,7,8], and difficulties in genetic engineering.

Research in PUFA biosynthesis in microalgae receives much attention also because microalgae are promising suppliers of omega 3-long chain PUFAs (ω3-LCPUFAs). ω3-LCPUFAs, e.g., eicosapentaenoic acid (EPA, C20:5*n*3) and docosahexaenoic acid (DHA, C22:5*n*3) are dietary-supplements for humans [9], with an increasing demand for production. The growing market has made it impossible to maintain a sustainable supply from the conventional source of fish oil. Some microalgal strains are well known to produce a high content of EPA and DHA [10], and thus are a potential alternative source of ω3-LCPUFAs. Therefore, understanding PUFA biosynthesis in microalgae will not only benefit the regulation of PUFA content for biodiesel application but also enable a stable supply of fatty acid dietary products.

Among eukaryotic microalgae, diatoms are well-established model organisms in terms of genomic and transgenic capabilities. A pennate diatom *Phaeodactylum tricornutum* has been used for basic research in both fuel and ω3-LCPUFA productions taking advantage of the available genome information [11] and easy transformation [12,13]. In *P. tricornutum*, thorough investigation revealed that EPA is synthesized as an end-product of PUFA synthesis via multiple metabolic networks, including the classical ω6-pathway, the classical ω3-pathway, a pathway relying on intermediates of both of these pathways and an alternative ω3-pathway involving Δ9-elongation and Δ8-desaturation [6]. However, the lipid production capacity of *P. tricornutum* is moderate, and, thus, this microalga may not be the best candidate lipid producer for practical application. 

*Fistulifera* sp. strain JPCC DA0580 is an oleaginous diatom screened from our marine microalgal culture collection [14]. Beneficial features of this strain for biodiesel production include high neutral lipid content (40%–60%, w*/*w), high growth rate, and low unsaturation degree of the accumulated lipids [14]. Recently multi-omic analyses, including whole genome [15], transciptome, lipidome, and proteome [16] analyses, of this oleaginous strain have been launched. Based on current genomic information, a transformation technique for this strain has also been established [17]. In this study, we determined the putative pathways for PUFA biosynthesis in *Fistulifera* sp. strain JPCC DA0580. From the genome database, genes encoding the desaturases and elongases of this diatom were identified. Subsequently, the subcelluar localization of these enzymes was predicted with a series of bioinformatic analyses. The predicted pathway was further confirmed by the detailed fatty acid profile which was determined by direct transesterification of the dried biomass and the subsequent GC-MS analysis. Finally, the transcriptomic results well explained the variation in the fatty acid profile over incubation time. The elucidation of the PUFA synthesis pathway in *Fistulifera* sp. JPCC DA0580 would facilitate gene modification in this strain to regulate PUFA content for production of ω3-LCPUFAs or for biodiesel application.

## 2. Materials and Methods

### 2.1. Culturing *Fistulifera* sp. Strain JPCC DA0580

The marine diatom, *Fistulifera* sp. strain JPCC DA0580, was isolated from the junction of the Sumiyo and Yakugachi Rivers, in Kagoshima, Japan (28°15′ N, 129°24′ E) [14]. *Fistulifera* sp. JPCC DA0580 was cultured in f/2 medium [18] (75 mg NaNO_3_, 6 mg Na_2_HPO_4_·2H_2_O, 0.5 µg vitamin B12, 0.5 µg biotin, 100 µg Thiamine HCl, 10 mg Na_2_SiO_3_·9H_2_O, 4.4 mg Na_2_-EDTA, 3.16 mg FeCl_3_·6H_2_O, 12 µg CoSO_4_·5H_2_O, 21 µg ZnSO_4_·7H_2_O, 0.18 mg MnCl_2_·4H_2_O, 70 µg CuSO_4_·5H_2_O, and 7 µg Na_2_MoO_4_·2H_2_O) dissolved per liter of artificial seawater. Cultures were aerated with sterile air at 25 °C under 140 µmol/m^2^/s continuous illumination. 

### 2.2. Pathway Prediction

Candidate genes involved in lipid metabolism were screened from the draft genome database of the *Fistulifera* sp. strain JPCC DA0580 [19], based on the Basic Local Alignment Search Tool Protein (BLASTP) search. Protein sequences with KEGG orthology (KO) number present in the Kyoto Encyclopedia of Genes and Genomes (KEGG) database or those described in published papers were used as query sequences. After screening, the candidate genes were submitted to InterProScan to identify protein signatures, and were subsequently annotated. In cases where gene annotations based on the protein signatures were ambiguous, phylogenetic tree analysis was also conducted to assess phylogenic relationships with diatom genes of known function. Finally, localization of proteins encoded by the annotated genes was predicted using several bioinformatics programs. TargetP [20] and HECTAR [21] were used for predicting general protein localization. Screening for endoplasmic reticulum (ER) or chloroplast-targeting peptides was performed by using SignalP [22]. When cleavage sites were predicted by SignalP, the presence of an ER retention signal (K(/D)-D(/E)-E-L) in the protein *C*-terminus was checked manually [23]. If proteins possessed no ER retention signals and contained F, W, Y, or L at the +1 position of cleavage sites, they were considered to be chloroplast-targeted proteins [24]. When proteins were predicted to be localized in the mitochondria by both TargetP and HECTAR, they were considered to be transported into mitochondria. In addition, Mitoprot [25] was used to detect mitochondrial-targeting peptides. If (i) the score of Mitoprot was >0.9 or (ii) the score of Mitoprot was >0.8, and mitochondrial localization was predicted by either TargetP or HECTAR, the protein was also considered to be transported into mitochondria. For proteins without signal, chloroplast-targeting, or mitochondrial-targeting peptides, peroxisome-targeting signals at the *C*-terminus (S(/A/C)-K(/R/H)-L(/M) or S-S-L) were checked manually [26]. A transmembrane prediction program (TMHMM) [27] was used to detect the presence of transmembrane regions. If transmembrane regions were predicted in proteins not containing other targeting peptides, those proteins were predicted to be localized in the ER. Proteins that conformed to none of the rules described above were predicted to be localized in the cytoplasm. 

### 2.3. Transcriptome Analysis

Total RNA was extracted according to a previously published procedure [14]. In brief, cells were collected by centrifugation (8500× *g*, 10 min, 4 °C) after 48, 96, and 144 h of cultivation. Cell pellets were frozen in liquid nitrogen and fractured using mortars and muddlers, and the resulting samples were treated with Plant RNA Isolation Reagent (Invitrogen Corp., Carlsbad, CA, USA) and incubated with DNase I (Takara Bio, Inc., Shiga, Japan) for 30 min at 37 °C. Subsequently, the total RNA was purified with the RNeasy Mini kit (Qiagen, Inc., Valencia, CA, USA). After purification, the quality of the isolated total RNA was evaluated using an Agilent 2100 Bioanalyzer (Agilent Technologies, Inc., Santa Clara, CA, USA).

For RNA sequencing, cDNA sequencing libraries were constructed using the mRNA-Seq Library Prep Kit (Illumina, Inc., San Diego, CA, USA) and the Small RNA Sample Prep Kit (Illumina, Inc., San Diego, CA, USA) according to the mRNA-Seq library preparation pre-release protocol rev.A (Illumina, Inc., San Diego, CA, USA). Briefly, poly A^+^ RNA was isolated from 10 µg of total RNA using oligo(dT) magnetic beads. The isolated poly A^+^ RNA was fragmented into small pieces under elevated temperature. After the fragmented RNA was treated with alkaline phosphatase (Takara Bio, Inc., Shiga, Japan), the 5′-end of the fragmented RNA was phosphorylated using T4 polynucleotide kinase (Takara Bio, Inc., Shiga, Japan). Subsequently, 5′- and 3′-adapters were ligated to the phosphorylated RNA using truncated T4 RNA ligase 2 (New England Biolabs, Inc., Ipswich, MA, USA). 

The adapter-ligated RNA was reverse transcribed using primers designed for the 3′-end adapter. The products from the reverse transcription reaction were amplified by 12 cycles of polymerase chain reaction (PCR). The resulting PCR products were size-fractionated by polyacrylamide gel electrophoresis to create a final cDNA sequencing library.

The cDNA sequencing libraries were sequenced using a Genome Analyzer IIx (Illumina, Inc., San Diego, CA, USA) according to the manufacturer’s instructions. Briefly, clusters were generated from the library on the surface of a flowcell by bridge amplification using a Cluster Station (Illumina, Inc., San Diego, CA, USA) and a Single-read Cluster Generation Kit v4 (Illumina, Inc., San Diego, CA, USA). Sequencing was performed using a Genome Analyzer IIx and a TruSeq SBS kit v5 (Illumina, Inc., San Diego, CA, USA). Illumina Sequence Control Software (SCS) v2.8 with Real Time Analysis (RTA) v1.8 was used for operation and data processing, including image analysis and base calling. 

Transcript abundances were expressed using the reads per kilobase of exon model per million mapped reads (RPKM) value [28]. Bowtie v0.12.7 [29] was used to align the obtained RNA-sequencing reads to the draft whole genome of *Fistulifera* sp. strain JPCC DA0580. ERANGE software v3.2 [28] was used to calculate RPKM values. Log_2_ fold changes in RPKM values (log_2_(RPKM_96h,144h_/RPKM_48h_)) were calculated to evaluate gene expression changes. RPKM_48h_ indicates the values obtained from the mRNA sample before starting TAG accumulation, while RPKM_96h_ and RPKM_144h_ indicate the values obtained from mRNA samples during the TAG accumulation process. Differentially expressed genes (log_2_ fold changes in RPKM values ≥1 or ≤−1) were selected for further investigation using Gene Ontology (GO) analysis. All sequences of the differentially expressed genes in the strain were submitted to InterProScan to identify protein signatures. GO terms assigned to specific InterPro signatures were used to classify the gene products. The GO terms were classified into functional groups based on “Plant GO Slim” categories using CateGOrizer [30].

### 2.4. Methyl Esterification and GC-MS Analysis

Lyophilized microalgal cells (25 mg) were used for methyl esterification. The dried microalgal biomass was transesterified by heating with 3 mL of 1.25 M HCl-methanol at 90 °C for 1 h. After methanolysis, the resulting FAMEs were extracted 3 times with *n*-hexane.

A Shimadzu GCMS QP2010 with a capillary column (0.22 mm × 25 m) and a Shimadzu HiCap-CBP 5 (Shimadzu, Kyoto, Japan) were used to determine the generated FAME compositions under the following conditions: column, FAMEWAX column (30 m, 0.25 mm ID, 0.25 µm); interface temperature, 240 °C; injection-port temperature, 240 °C; ion-source temperature, 260 °C; and helium gas pressure, 60.4 kPa. Oven temperatures were programmed to 140 °C for 5 min, then to 240 °C, increasing at 4 °C/min, and finally to hold at 240 °C for 10 min. FAMEs were identified by comparing the peak retention times and mass spectra of the samples with those of a standard fatty acid mixture. The GC-MS measurements were repeated three times for each sample.

## 3. Results

### 3.1. Characteristics of the Enzymes Necessary for PUFA Synthesis

From the *Fistulifera* sp. strain JPCC DA0580 genome, we determined putative gene sets involved in the PUFA synthesis pathway. *Fistulifera* sp. possesses a complete gene set for EPA synthesis (*i.e.*, Δ9-, Δ12-, Δ6-, Δ5-, ω3-desaturase, and Δ6-elongase). Subcellular localization of the enzymes encoded by the determined genes was also predicted by a series of bioinformatics processes. The characteristics of the putative enzymes (e.g., localization, catalytic motif, and transmembrane domains) are summarized in Table 1. The same enzymes encoded in the genomes of other diatoms, *i.e.*, *P. tricornutum* [11] and *Thalassiosira pseudonana* [31], were also listed for comparison. Because the putative genes were determined by BLAST search based on previously identified desaturases or elongases in other organisms, it is possible that other desaturases and elongases may remain undiscovered in the *Fistulifera* sp. genome if they happen to have low sequence similarity to the conventional enzymes.

All desaturases determined in this study possess 3 histidine boxes, a common active site of desaturases involved in the coordination of the di-iron center [32]. Similar to the Δ6- and Δ5-desaturases (the so-called “front-end desaturases”) found in other organisms, those of *Fistulifera* sp. strain JPCC DA0580 also have a characteristic third histidine box, which begins with a glutamine residue instead of a histidine [33]. As we reported previously, Δ9-desaturases were divided into 2 groups: ER- and chloroplast-desaturases [34]. In this study, we determined that the Δ12-desaturases also exhibit the same characteristics. In particular, G13618 (encoded by AB858393 (*g13618*)) and G3281 (encoded by AB858398 (*g3281*)) were predicted to localize at the ER, while G13836 (encoded by AB858392 (*g13836*)) and G14210 (encoded by AB858391 (*g14210*)) were at the chloroplast. It should be noted that chloroplast Δ6-desaturases have not been predicted to be expressed by the three diatoms listed in Table 1; thus, the subcellular compartments in diatoms required for the desaturation of C16:2 into C16:3 are still unclear.

**Figure 1:**
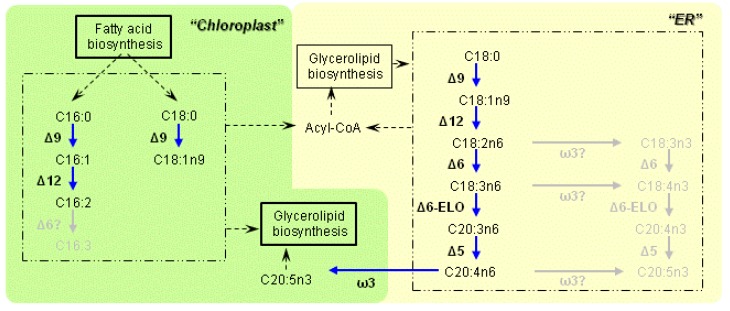
The fatty acid desaturation pathway in *Fistulifera* sp. strain JPCC DA0580, as proposed by this study. Desaturation and elongation reactions catalyzed by specific enzymes are indicated by blue arrows. Potential exchange reactions are indicated by dashed arrows. The pathways in gray letters are predicted to be inactivated in this strain due to the absence of the enzymes (interrogation marks).

Furthermore, the localization prediction of all the desaturases and elongases involved in EPA synthesis in *Fistulifera* sp. revealed an unexpected feature that the putative ω3-desaturases localize at the chloroplast in this strain but the ER (Figure 1 and Table 1). In addition to the sequence features which were considered in our pipeline to predict protein localization (e.g., signal/transit peptides and transmembarane domains), there is another feature supporting the localization of the ω3-desaturases in the chloroplast that they have a serine/threonine-rich region after the signal peptides in the *N*-termini of these proteins [35]. In contrast to *Fistulifera* sp. strain JPCC DA0580, the ω3-desaturases of the other two sequenced diatoms were predicted to localize at the ER. However, other Δ9-, Δ12-, Δ6-, and Δ5-desaturases were predicted to localize at the ER in *Fistulifera* sp. strain JPCC DA0580. 

**Table 1:** Characteristics of desaturases and elongases found in the genome of *Fistulifera* sp. strain JPCC DA0580.

Desaturase	Organisms	Accession No.	Predicted localization	Cytochrome b5 domain	Conserved histidine boxes			No. of predicted TMHs	
					First	Second	Third	TMHMM	HMMTOP
Δ12 (*1) desaturase	*Fso*	AB858393 (*g13618*)	ER (*4)	−	HECGH	HAKHH	HVVHH	5	6
		AB858398 (*g3281*)	ER (*4)	−	HECGH	HAKHH	HVVHH	5	6
		AB858392 (*g13836*)	Chloro	−	HECGH	HAVHH	HVAHH	5	5
		AB858391 (*g14210*)	Chloro	−	HECGH	HAVHH	HVAHH	5	5
	*Ptr*	XM_002186103	ER (*4)	−	HECGH	HAKHH	HVVHH	4	5
		XM_002182796	Chloro	−	HECGH	HAVHH	HVAHH	5	5
	*Tps*	XM_002292035	ER (*4)	−	HECGH	HAKHH	HVAHH	4	2
		XM_002288140	Chloro	−	HECGH	HAVHH	HVAHH	4	3
ω3 desaturase	*Fso*	AB858396 (*g8143*)	Chloro	−	HDAGH	HKKHH	HVIHH	6	4
		AB858395 (*g9395*)	Chloro	−	HDAGH	HKKHH	HVIHH	6	4
	*Ptr*	XM_002185462	ER (*4)	−	HDAGH	HLKHH	HLVHH	6	6
	*Tps*	XM_002291021	ER (*4)	−	HDAGH	HRKHH	HVVHH	2	2
Δ6 desaturase (*2)	*Fso*	AB858389 (*g18158*)	ER (*4)	+	HDFLHH	WKNKHNGHH	QVDDHHLFP	4	4
		AB858388 (*g18269*)	ER (*4)	+	HDFLHH	WKNKHNGHH	QVDDHHLFP	4	4
	*Ptr*	XM_002182865	ER	+	HDFLHH	WKNKHNGHH	QVDDHHLFP	2	3
	*Tps*	XM_002291493	ER (*4)	+	HDFLHH	WKNKHNGHH	QVDDHHLFP	4	4
Δ5 desaturase (*2)	*Fso*	AB858387 (*g19653*)	ER (*4)	+	HDANH	WQEQHWTHH	QVEHHLFP	1	6
		AB858397 (*g7119*)	ER (*4)	+	HDANH	WQEQHWTHH	QVEHHLFP	3	6
	*Ptr*	XM_002185696	ER	+	HDANH	WQEQHWTHH	QVEHHLFP	4	5
		XM_002182822	ER (*4)	+	HDANH	WIQKHWTHH	QVEHHLFP	4	6
	*Tps*	XM_002296831	ER (*4)	+	HDANH	WLAQHWTHH	QVEHHLFP	5	8
		XM_002288806	ER (*4)	+	HDANH	WMAQHWTHH	QVEHHLFP	4	6
**Elongase**	**Organisms**	**Accession No.**	**Predicted localization**		**Conserved elongase motif**			**No. of predicted TMHs**	
								**TMHMM**	**HMMTOP**
Δ6 poly unsaturated elongase (*3)	*Fso*	AB858394 (*g11153*)	ER (*4)		QLSFLHVYHH			5	7
		AB858390 (*g17615*)	ER (*4)		QLSFLHVYHH			5	7
	*Ptr*	XM_002180392	ER (*4)		QLSFLHVYHH			7	7
		XM_002182520	ER (*4)		QLSFLHVYHH			5	6
	*Tps*	XM_002288445	ER (*4)		QLSFLHVYHH			7	7

(*1) Conserved histidine boxes are determined with the reference [35]; (*2) Conserved histidine boxes are determined with the reference [36]; (*3) Conserved elongase motifs are determined with the reference [33]; (*4) Desaturases are designated as predicted transmembrane proteins localized at the ER but not containing an ER-targeting signal peptide.

### 3.2. Variation of the Fatty Acid Composition in *Fistulifera* sp. during Cultivation

**Figure 2:**
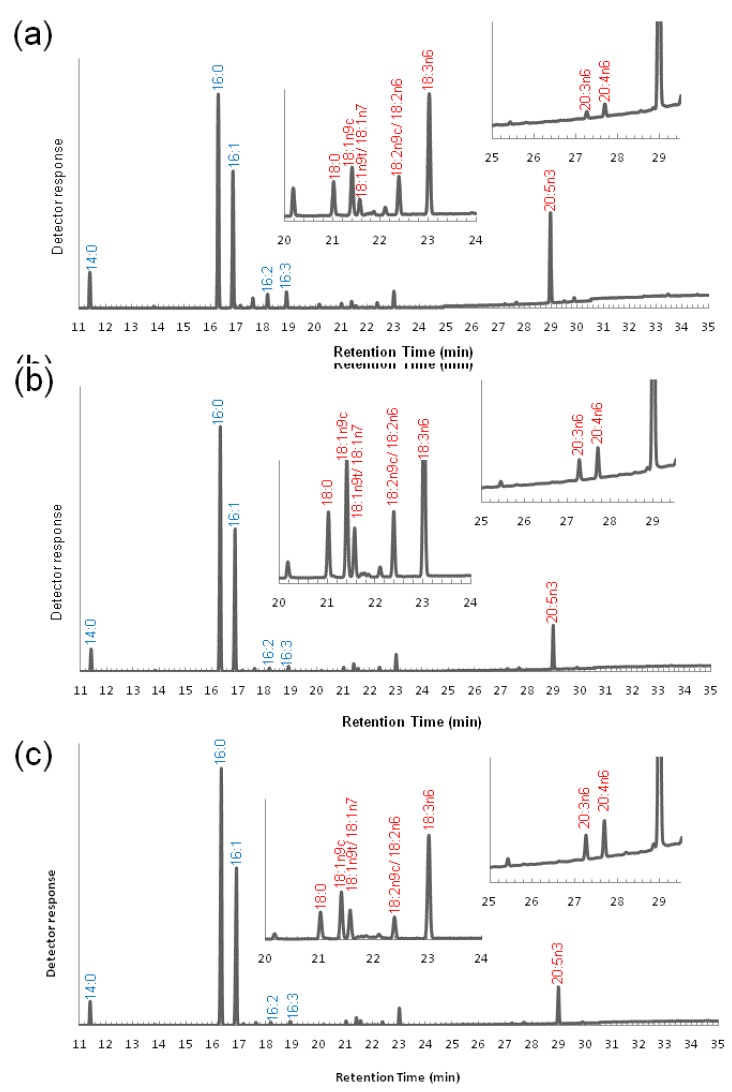
Gas chromatographic analysis of total fatty acid methyl esters (FAMEs) produced from microalga biomass of *Fistulifera* sp. strain JPCC DA0580 at (**a**) 48 h, (**b**) 96 h and (**c**) 144 h of cultivation. Fatty acids in red letters are precursors for EPA synthesis.

The composition of FAME produced from the dried cells was analyzed during cultivation with the conventional f/2 medium (Figure 2, see also Supplementary Table S1). The methyl ester forms of C16:0 and C16:1 were dominant throughout the analytical points (*i.e.*, 48 h, mid-log phase; 96 h, late-log phase and 144 h, stationary phase), and both increased during cultivation. Specifically, the proportion of C16:0 was 29.8% ± 0.2%, 37.3% ± 0.2%, and 36.5% ± 0.0% of total FAMEs at 48 h, 96 h, and 144 h, respectively, while C16:1 was 36.3% ± 0.3%, 42.4% ± 0.3%, and 45.9% ± 0.0%, at the same time-points. C16:2 and C16:3 were detectable in trace amounts. C20:5n3 (EPA) was the major PUFA methyl esters present, and its content decreased with incubation time (17.0% ± 0.1%, 8.3% ± 0.1%, and 6.6% ± 0.0% of total FAMEs at 48 h, 96 h, and 144 h, respectively). These variations over the incubation time, such as the increase of the C16:1 and C16:0 and the decrease of the C20:5, were also observed in our previous work [37], and thus considered as the representative characters of *Fistulifera* sp. A series of C18 and C20 fatty acid precursors for EPA synthesis (Figure 2) was confirmed, although the peaks were very small. It should be noted that all of the C18 and C20 fatty acids detected in this study were ω6-fatty acids (C18:3*n*6, C20:3*n*6 and C20:4*n*6), and no ω3-fatty acids (C18:3*n*3, C18:4*n*3 and C20:4*n*3) were detected, except for C20:5n3 (EPA).

### 3.3. Transcriptome Analysis of the Enzymes for Fatty Acid Desaturation

**Figure 3:**
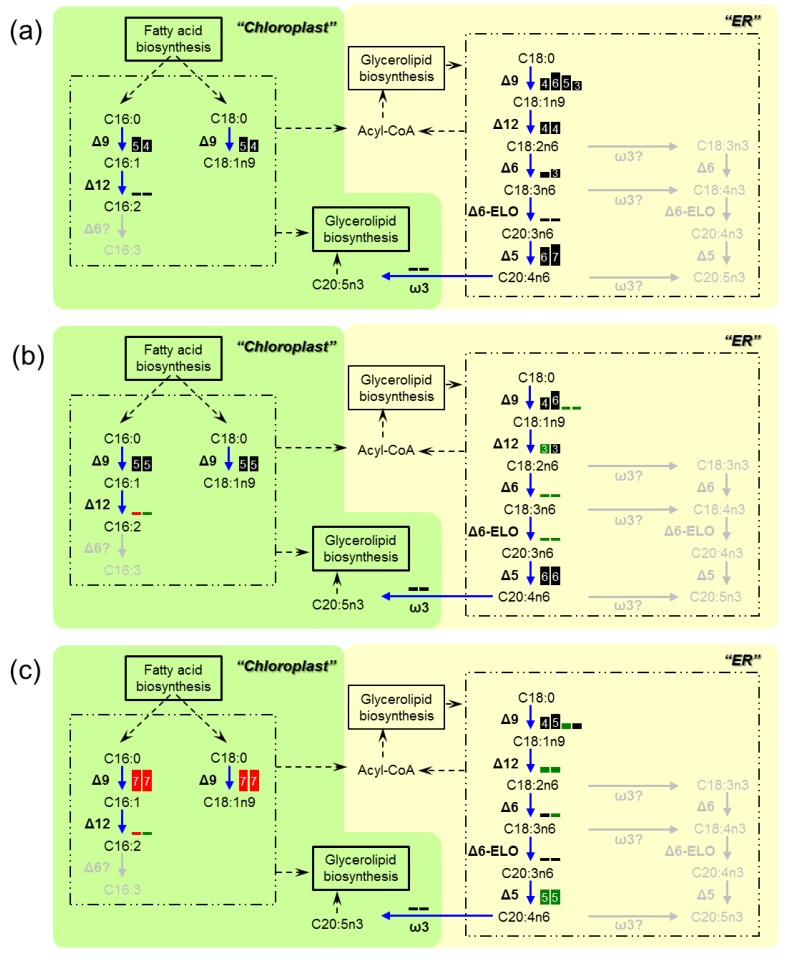
Genetic changes in the fatty acid desaturation pathway in *Fistulifera* sp. strain JPCC DA0580, as proposed by this study. Transcription activities at (**a**) 48 h, (**b**) 96 h and (**c**) 144 h of cultivation are displayed as boxes. Each box represents an enzymeisoform. Sizes of the boxes and numbers in the boxes represent magnitudes of the RPKM values (3: (8)~16, 4: ~32, 5: ~64, 6: ~128, 7: ~256, 8: ~512, 9: ~1024, 10: 1024~). Red boxes indicate that Log_2_ fold changes in RPKM values (96/48 h or 144/48 h) were ≥1. Green boxes show that log_2_ fold changes in RPKM values were ≤−1.

To determine the genetic changes responsible for the FAME variation, we also analyzed the expression of the desaturases and elongases after 48 h, 96 h and 144 h of cultivation (Figure 3). During the incubation period, the Δ9-desaturases in the chloroplast, which could catalyze the conversion of C16:0 into C16:1 and C18:0 into C18:1, were the only enzymes that were up-regulated with high RPKM values. The subsequent reaction, in which C16:1 is converted into C16:2, seems to be significantly inactivated, as the Δ12-desaturases in the chloroplast had low RPKM values. Additionally, overall down-regulation of the ER-desaturases and elongases was confirmed.

## 4. Discussion

The genomic analysis suggested that *Fistulifera* sp. strain JPCC DA0580 has two distinct pathways for fatty acid desaturation in the ER and chloroplast, as has been reported for other diatoms. The functions of diatom desaturases have been well studied in the diatom *P. tricornutum*, where it was demonstrated that the ER-Δ12-desaturase (encoded by XM_002186103) is highly specific to C18:1*n*9 while the chloroplast-Δ12-desaturases (encoded by XM_002182796) is highly specific to C16:1 [35]. The amino acid sequences of both ER- and chloroplast-desaturases of the strain JPCC DA0580 were substantially similar to their counterparts in *P. tricornutum*. The protein G13618 and G3281 ER-desaturases showed 91% similarity to the ER-Δ12-desaturase in *P. tricornutum*, while the G13836 and G14210 chloroplast-desaturases showed 95% and 93% similarity to the chloroplast-Δ12-desaturases in *P. tricornutum*. Therefore, it is reasonable to consider that the Δ12-desaturases in the ER and chloroplast of *Fistulifera* sp. would have similar substrate preferences as their counterparts in *P. tricornutum*. These results indicate that desaturation of C18 and C16 fatty acids mainly take place in the ER and chloroplast, respectively. Altogether, we have established a putative metabolic map for PUFA synthesis in *Fistulifera* sp. strain JPCC DA0580 (Figure 1).

One striking feature of the established metabolic map is the absence of ω3-desaturases in the ER, and their presence in the chloroplast. This characteristic localization has not been observed even in two other sequenced diatoms (Table 1). During EPA synthesis, the ω3-desaturase introduces a double bond at the ω3 position in the fatty acid moiety. This enzyme catalyzes the first step for EPA synthesis in the ω3 pathway, while playing a role at the final step of synthesis in the ω6 pathway (Figure 1). As no ω3-desaturase has been predicted to localize at the ER, we hypothesize that *Fistulifera* sp. strain JPCC DA0580 would utilize the ω6 pathway where C20:4n6 (an immediate precursor of EPA) is synthesized at the ER, with the final step in the synthesis of EPA (C20:5*n*3) being carried out by the ω3-desaturases in the chloroplast (Figure 1). This hypothesis is further supported by the revealed FAME profile, in which the presence ω6-fatty acids (C18:3*n*6, C20:3*n*6, and C20:4*n*6) was confirmed, while no ω3-fatty acids (C18:3*n*3, C18:4*n*3, and C20:4*n*3) were detected as precursors of EPA synthesis (Figure 2).

The proposed pathway depicted in Figure 1 resembles that of the red algae *Porphyridium cruentum* [38] rather than that of the pennate diatom *P. tricornutum*, which synthesizes EPA through both pathways in a cross-sectional manner [6]. This finding strongly suggests that the synthesis pathways for PUFAs (like EPA) are different even between the same pennate diatoms. In other words, molecular engineering efforts to increase or decrease PUFA content must target the enzymes in the appropriate pathway of each organism of interest.

The critical advantages of *Fistulifera* sp. strain JPCC DA0580 as a feedstock for biodiesel production include, not only the high capacity of oil accumulation [14], but also a low content of PUFA methyl esters. However, the underlying molecular mechanisms behind the beneficial low PUFA content have remained unknown. In this study, we sought clues to elucidate these underlying mechanisms via transcriptome analysis to detect key genes involved in fatty acid desaturation. As a result, the expression of the ER -enzymes in the ω6 pathway (*i.e.*, Δ9-, Δ12-, Δ6-, and Δ5-desaturases and Δ6-elongases) was found to be globally down-regulated during incubation (Figure 3). This transcriptional regulation can be a reason for the decrease in EPA. Addition to EPA, the fatty acid analysis showed that the percentage of C18:2*n*6 and C20:4*n*6 also showed slight decrease, while other fatty acids involved in the ω6 pathway were stable over culture time (Supplementary Table S1). All those fatty acids were present in really low proportion. Low content of C16:2 and C16:3 could be another factor contributing to the low PUFA content in *Fistulifera* sp. strain JPCC DA0580, as other diatoms possess substantial amounts of these fatty acids [10]. These findings could also be explained by the variation in the expression of corresponding genes. As opposed to the ER-Δ9-desaturases, Δ9-desaturases of the chloroplast were up-regulated and maintained high RPKM values. However, chloroplast-Δ12-desaturases, predicted to be specific to C16 fatty acid based on homology analysis, were expressed at extremely low levels. These expression patterns could result in the increase of dominant C16:1 during the incubation, and also contribute to the decreased content of C16:2 and subsequent C16:3. We conclude that unique transcriptional activities in the fatty acid desaturation pathways drives establishment of the characteristically low PUFA methyl ester content in *Fistulifera* sp. strain JPCC DA0580.

## 5. Conclusions

Based on the sequence analysis of genes which were potentially involved in the EPA synthesis, and the detailed FAME profile, we putatively determined the biosynthetic pathways for fatty acid desaturation in the oleaginous diatom, *Fistulifera* sp. strain JPCC DA0580. The putative pathways and their transcriptional activities were very consistent with the FAME profiles revealed by the GC-MS analysis. Interestingly, the data suggested that the metabolic pathway for EPA synthesis in *Fistulifera* sp. strain JPCC DA0580 was likely to be different from that in the pennate diatom *P. tricornutum*. Results from this study will be valuable to enable future metabolic engineering in order to increase EPA synthesis for commercial use, as well as to decrease poly-unsatulated FAMEs in biodiesel application. 

## Supplementary Files

Supplementary File 1 Supplementary Material (PDF, 16 KB)
