# Supplementary material for: Biosynthesis of Polyunsaturated Fatty Acids in the Oleaginous Marine Diatom Fistulifera sp. Strain JPCC DA0580
Source: Mar Drugs. 2013 Dec 11;11(12):5008–23. doi: 10.3390/md11125008 (PMC3877899; doi:10.3390/md11125008)
Supplement: Supplementary File 1 — Supplementary Material (PDF, 16 KB) [file marinedrugs-11-05008-s001.pdf]

Supplementary Material

Table S1. The fatty acid composition in *Fistulifera* sp. over incubation time GC-MS detection was repeated three times.

Name	48 h-mol%	96 h-mol%	144 h-mol%
14:0	4.5 ± 0.1	3.0 ± 0.0	2.9 ± 0.0
16:0	29.8 ± 0.2	37.3 ± 0.2	36.5 ± 0.0
16:1	36.3 ± 0.3	42.4 ± 0.3	45.9 ± 0.0
16:2*n*4	2.5 ± 0.0	0.7 ± 0.0	0.6 ± 0.0
16:3*n*4	3.0 ± 0.4	1.1 ± 0.0	0.8 ± 0.0
18:0	0.6 ± 0.0	0.6 ± 0.0	0.6 ± 0.0
18:1*n*9	1.1 ± 0.0	1.4 ± 0.0	1.3 ± 0.0
18:1*n*7	0.3 ± 0.0	0.5 ± 0.0	0.8 ± 0.0
18:2*n*6	0.9 ± 0.0	0.7 ± 0.0	0.6 ± 0.0
18:3*n*6	2.7 ± 0.0	2.9 ± 0.1	2.8 ± 0.0
20:3*n*6	0.2 ± 0.0	0.3 ± 0.0	0.2 ± 0.0
20:4*n*6	0.4 ± 0.0	0.4 ± 0.0	0.3 ± 0.0
20:5*n*3	17.0 ± 0.1	8.3 ± 0.1	6.6 ± 0.0
Others	0.7 ± 0.0	0.4 ± 0.0	0.3 ± 0.0

© 2013 by the authors; licensee MDPI, Basel, Switzerland. This article is an open access article distributed under the terms and conditions of the Creative Commons Attribution license (http://creativecommons.org/licenses/by/3.0/).
